# Feasibility of using digital confocal microscopy for cytopathological examination in clinical practice

**DOI:** 10.1038/s41379-021-00925-4

**Published:** 2021-10-09

**Authors:** Savitri Krishnamurthy, Kechen Ban

**Affiliations:** 1 Department of Pathology and Laboratory Medicine, The University of Texas, MD Anderson Cancer Center, Houston, TX USA; 2 Department of Neurosurgery Research, The University of Texas, MD Anderson Cancer Center Houston, Houston, TX USA

**Keywords:** High-throughput screening, Biological techniques

## Abstract

Optical imaging modalities are emerging as digital microscopy tools for tissue examination. The investigation of these techniques for potential applications in anatomic pathology practice has focused primarily on surgical pathology and has not included cytopathological specimens. We evaluated the feasibility of using digital confocal microscopy (CM) to examine cytopathological specimens. Smears and cell suspensions collected in RPMI solution were prepared from tissue scrapes obtained from surgical resections of breast, lung, liver, and kidney. Air-dried smears and cell pellets obtained from centrifugation of the cell suspensions were stained with 0.6 mM acridine orange and imaged with a CM platform. After completion of imaging, the smears were stained with Diff-Quik (DQ), and cell pellets were routinely processed, embedded in paraffin wax, cut, and stained with hematoxylin and eosin (H&E). We evaluated the mean time to acquire digital CM images; quality of images based on the extent of tissue recognition (0%, grade 0; 1–19%, grade 1; 20–50%, grade 2; >50%, grade 3); comparison of images with DQ- and H&E-stained specimens; and ability to make specific diagnoses. We imaged 91 smears and 52 cell pellets and acquired digital CM images within 2–3 min, with 92% and 88% of images, respectively, from smears and cell pellets showing grade 3 quality. On the basis of CM images, 8 smears (9%) and 7 cell pellets (14%) were categorized as benign, and 83 (91%) and 45 (88%), respectively, as malignant. Specific diagnoses were made by using digital CM images of smears and cell pellets that matched accurately with corresponding DQ- and H&E-stained preparations. The results of our first feasibility study clearly indicated the utility of CM as a next-generation digital microscopy tool for evaluating cytology specimens. Prospective clinical studies are warranted for validating our findings for potential incorporation into cytopathological clinical practice.

## Introduction

Optical imaging modalities are currently being investigated for in vivo and ex vivo visualization of human tissues. These modalities use light in the visible and adjacent spectra and exploit the special properties of photons interacting with labeled or unlabeled components in tissues, thereby enabling the acquisition of tissue images. Ex vivo tissue imaging, using a variety of optical imaging techniques, can be appealing to practicing pathologists because these techniques allow the acquisition of digital images that permit recognition of tissues, similar to the way in which these tissues are recognized with use of the light microscope^1^. However, in contrast to traditional bright field pathological examination that requires elaborate tissue preparation, including staining with various dyes, optical imaging modalities are inherently digital and allow the recognition of tissues with minimal or no tissue preparation. These modalities are essentially optical sectioning digital microscopy techniques that can be exploited for evaluation of tissues. A distinct advantage of these techniques is the possibility of real-time evaluation of digital images either at the site of procurement or remotely from any location. In addition, the digital images can be easily stored, retrieved, integrated into electronic health records, and used for building artificial intelligence tools for image interpretation.

In recent years, rapid advancements in the fields of biophotonics, computer science, and instrumentation technology have enabled the development of optical imaging platforms that can be used for ex vivo imaging of tissues that are encountered in surgical pathology clinical practice. The optical principle behind the different types of ex vivo tissue imaging modalities is variable. Techniques based on confocal microscopy (CM), optical coherence tomography (OCT), full-field OCT (FF-OCT), light sheet microscopy, structured illumination microscopy, stimulated Raman spectroscopy (SRS), nonlinear microscopy, and microscopy using ultraviolet surface excitation (MUSE) have been used to build optical imaging platforms. Optical imaging platforms based on CM, OCT, FF-OCT, and SRS alone are currently available commercially for ex vivo tissue imaging. Currently, there is much interest in evaluating the potential of these imaging modalities for several applications pertinent to surgical pathology clinical practice. These applications include rapid evaluation of core needle biopsies and other types of small tissue biopsies, intraoperative assessment of tissues including margin assessment of surgical resections, and evaluation of the quality of tissues procured for biobanking. The feasibility of some of these relevant applications of optical imaging in surgical pathology has been demonstrated in recent years^2–6^.

Although there is strong interest in investigating the various types of optical imaging modalities for potential applications in surgical pathology, the feasibility and utility of these techniques for the evaluation of cytological specimens, including smears and cell pellets, that are routinely encountered in cytopathological practice have not been investigated. In the present study, we sought to evaluate the feasibility of using digital CM to examine cytopathological specimens with use of smears and cell pellets prepared from tissue scrapes obtained from normal and tumor tissue from surgical resection specimens.

## Materials and methods

For our prospective study conducted under an Institutional Review Board–approved protocol, we used residual tissue obtained from surgical resections after completion of intraoperative assessment and tissue sampling for diagnostic pathology reporting. Tissue scrapes were obtained from tissue fragments of tumor or normal tissue from a variety of organs including lung, liver, breast, and kidney and were used to prepare air-dried smears on standard glass slides and/or were collected as cell suspension in RPMI solution. The two different preparations of the tissue scrapes were prepared to recapitulate direct smears and cell suspensions that are used for cell block preparation in routine cytopathological practice. The air-dried smears were used as such for CM, whereas the RPMI solution with the cell suspension was centrifuged at 1500 rpm to generate a cell pellet that was lifted with a spatula and placed onto a standard glass slide for CM.

### CM imaging of smears and cell pellets

The air-dried smears and cell pellets were stained with 0.6 mM acridine orange for ~10 s and then imaged immediately with use of a CM platform (Vivascope 2500 RSG4; Caliber ID, Inc, Rochester, New York, USA) The CM platform was designed specifically for ex vivo imaging of fresh biological tissue specimens. The platform includes a diode laser that operates at wavelengths of 488 and 785 nm, a 550-nm bandpass filter, a maximum illumination power of 5 mW, and an oil immersion objective lens with a numerical aperture of 0.9 and original magnification of x 40. At these settings, the lateral resolution was 1.0 µm, and the axial resolution was less than 5.0 µm. Images were acquired at 9 frames per second, and the composite image was 2.0 cm at the greatest diameter. The CM scanning of smear and cell pellet was performed with use of light at 488 and 785 nm, respectively to obtain confocal reflectance microscopy and confocal fluorescence microscopy images simultaneously. There was no need to program the area for imaging because this was built in the imaging platform. The tissue was brought to focus and then imaged to acquire the digital confocal microscopy images. The two types of images were overlaid and pseudocolored automatically pink and blue to mimic stained smears and tissue sections. The smear that was used for CM imaging was subsequently air-dried and stained by the Diff-Quik (DQ) method for conventional cytopathological examination. After acquisition of the digital CM images, the cell pellet was collected into a vial with an alcohol/formalin mixture, which was centrifuged at 1500 rpm for 5 min to recreate the cell pellet that was originally used for CM imaging. The final cell pellet was lifted with a spatula, wrapped in Shark Skin Filter Paper (Cytiva Whatman), and immersed into 10% formalin. The cell block prepared from the cell pellet was routinely processed to generate formalin-fixed and paraffin-embedded tissue blocks that were cut to a 5-µm thickness and stained by H&E for histopathological examination.

**Fig. 1:**
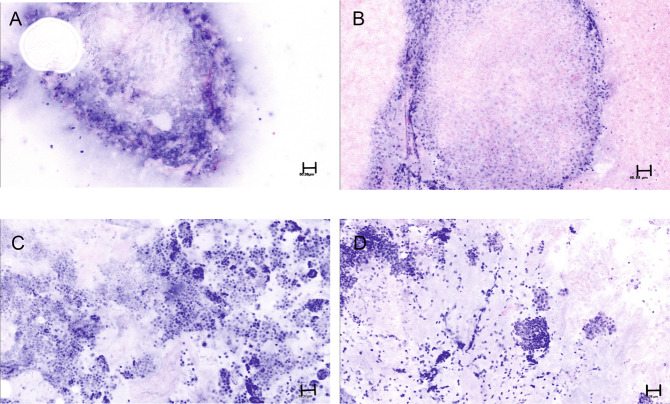
Criteria for grading digital confocal microscopy (CM) images. Representative examples of digital confocal microscopy images belonging to the 4 grades that exemplify the criteria used for grading into the four groups are illustrated. **A** Grade 0 (0%: Pseudocolored digital CM image where 0% of the tissue is recognized. **B** Grade 1; Less than 5% of the tissue is recognized in the digital CM image. **C** Grade 2: ~30% of the tissue is recognized in this digital CM image. **D** Grade 3: The entire tissue can be recognized in this digital CM image with optimal resolution for interpretation.

The digitally pseudocolorized CM images of smears and cell pellet, were examined in detail at various magnifications corresponding to 1x to 40x of a light microscope either at the site of acquisition or remotely with use of TeamViewer software, version 14.2.8352. The grading, categorization, and specific diagnosis of the digital CM images were performed by an experienced cytopathologist (SK). The quality of each CM image was graded according to the percentage of smear or cell pellet that could be recognized with optimal resolution to make a diagnosis (0%, grade 0; 1–19%, grade 1; 20–50%, grade 2; >50%, grade 3); the appearance of each CM image was compared with that of the corresponding DQ-stained smear or H&E-stained cell block. Representative examples of grade 0, grade 1, grade 2 and grade 3 quality of the digital CM images is shown in Fig. 1A–D. On the basis of cytomorphological features, the digital CM images were categorized as benign, atypical, suspicious, or malignant, and the ability to make specific diagnoses was determined. The CM images of the smears and cell pellets were compared with the DQ-stained smears and H&E-stained cell block sections.

## Results

We imaged 91 smears (27 breast, 27 liver, 23 lung, and 14 kidney) 52 cell pellets (26 breast, 11 liver, 11 lung, and 4 kidney). Each image was acquired within 2–3 min. The digital confocal images could be viewed in real time or at any time either at the site of procurement or remotely. The quality of the CM images of the 91 smears, graded 0–3 according to the percentage of smear that could be recognized with optimal resolution to make a diagnosis, was grade 3 (92.3% recognized) in 84 specimens and grade 2 (7.6% recognized) in 7 specimens; the quality of the CM images of the 52 cell pellets, graded 0–3, was grade 3 (88.2% recognized) in 45 specimens and 2 (11.7% recognized) in 6 specimens.

The specimens graded as 3 showed optimal resolution that allowed visualization of almost all of the cytological material and permitted accurate interpretation of the smears and cell pellets. The grade 2 images showed uneven staining of the cells in the centers of some of the clusters and fragments, but this did not compromise the overall interpretation of the specimen. The CM images demonstrated the nuclear and cytoplasmic contrast and resolution necessary to categorize the specimens. The pseudocolored digital CM images of the smears and cell pellets showed a darker shade of blue color of the nucleus and a much lighter blue color or a pink color of the cytoplasm of the cellular constituents. A distinct pink staining of the cytoplasm contrasting with the blue digital staining of the nucleus was noted in many but not all cases. Specific benign and malignant diagnoses could be made based on the recognition of conventional cytomorphological features in the digital CM images of smears and cell pellets, which matched accurately with the findings in DQ- and H&E-stained specimens. The amount of viable cellular content and its distinction from necrotic material could also be clearly appreciated in the specimens.

Based on the cytomorphological features, 8 (8.8%) of 91 smears were categorized as benign and 83 (91.2%) as malignant. The 52 cell pellets were categorized as benign in 7 cases (13.7%) and as malignant in 44 cases (86.3%). The integrity of the smears was well preserved for subsequent DQ staining that could be used for conventional cytopathological examination. Similarly, the formalin-fixed and paraffin-embedded cell blocks prepared from the imaged cell pellets showed optimal preservation of the cells based on evaluation of the H&E-stained tissue sections of the cell blocks. The cytomorphological features exhibited in the digital CM images of smears and cell pellets obtained from breast, lung, kidney, and liver tissue aided in the accurate recognition of the cytological material. Table 1 summarizes the specimen source of the smears and cell pellets, cytological categorization, and specific diagnosis that could be made on the digital CM images.

**Table 1:** Specimen source and cytological diagnosis of smears and cell pellets evaluated by digital confocal microscopy.

Specimen source		Cytological diagnosis
Smears	Benign	Malignant
Breast (*n* = 27)		Ductal carcinoma (26)
		Spindle and epithelioid tumor (1)
Lung (*n* = 23)		Squamous carcinoma (2)
		Adenocarcinoma (8)
		PDNSCLC (3)
		Neuroendocrine tumor (2)
		SCMT (8)
Kidney (*n* = 14)	Oncocytoma (1)	Renal cell carcinoma (13)
Liver (*n* = 27)	Benign hepatocytes (7)	Adenocarcinoma (17)
		Necrosis with rare ^*^PDC (3)
Cell pellets		
Breast (*n* = 26)	Benign ductal cells (2)	Ductal carcinoma (24)
Lung (*n* = 11)		Squamous carcinoma (1)
		Adenocarcinoma (2)
		PDNSCLC (4)
		SCMT (4)
Kidney (*n* = 4)		Renal cell carcinoma (4)
Liver (*n* = 11)	Benign hepatocytes (5)	Adenocarcinoma (4)
		Necrosis rare PDC (2)

*PDNSCLC* poorly differentiated non–small cell lung carcinoma, *SCMT* spindle cell malignant tumor, *PDC* poorly differentiated carcinoma.

### Breast

**Fig. 2:**
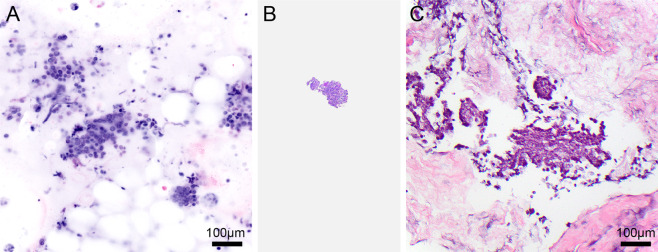
Digital confocal microscopy image of a cell pellet from a breast specimen. Pseudocolored digital (CM) image (**A**) of a cell pellet showing fragments of tumor cells with features of ductal carcinoma of breast, which resemble the hematoxylin and eosin–stained tissue section (**C**) of the cell block (**B**) prepared from the imaged tissue.

The digital CM images from 2 cell pellets showed few cohesive clusters of ductal epithelial cells without atypia with associated myoepithelial cells. The overall features were consistent with sampling of benign breast tissue. The digital CM images of all of the 27 smears and 24 cell pellets prepared from breast tissue exhibited increased cellularity including loosely cohesive clusters and individually dispersed atypical epithelial cells. There was no evidence of a second population of myoepithelial cells associated with the clusters or the presence of naked nuclei in the background. The constituent cells showed varying grades of nuclear atypia including the presence of nuclear membrane irregularity, prominent nucleolus, coarse nuclear chromatin, and mitotic figures. The amount of cytoplasm in the tumor cells varied from scant to moderate. Figure 2A–C shows a digital CM image of a cell pellet recognized as ductal carcinoma, with a corresponding H&E stain of the cell block prepared from the imaged tissue. A single smear showed spindle and epithelioid tumor cells with evidence of necrosis in the background. The overall cytomorphological findings in the digital CM images permitted the specific diagnosis of ductal carcinoma in 26 smears, spindle and epithelioid tumor in 1 smear, benign breast tissue in 2 cell pellets, and ductal carcinoma in 24 cell pellets. These findings accurately matched those noted in the corresponding DQ-stained smear and H&E-stained cell blocks.

### Lung

**Fig. 3:**
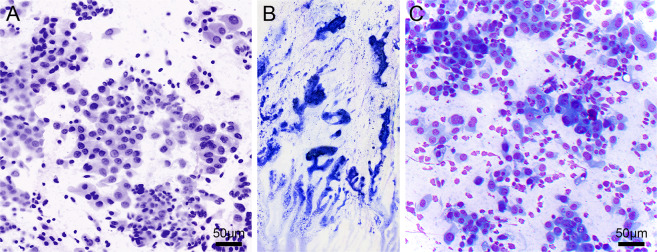
Digital confocal microscopy image of a smear from a lung specimen. Pseudocolored digital CM image (**A**) of a smear from a lung tumor showing features of a poorly differentiated non–small cell carcinoma, which appears similar to the findings in **C** in the corresponding Diff Quik (DQ)-stained smear (**B**).

Two smears and 1 cell pellet showed sheets of polygonal cells with a hyperchromatic nucleus and dense cytoplasm that included tumor cells with organophilic cytoplasm in 2 cases indicating keratinization in the tumor cells. The cytomorphological features in these cases allowed recognition as squamous carcinoma. Eight smears and 2 cell pellets showed increased cellularity including clusters and single cells with varying degrees of nuclear atypia, including the prominent nucleolus and mitotic figures. The presence of moderate amounts of delicate or vacuolated cytoplasm in the tumor cells together with the arrangement of tumor cells as three-dimensional clusters allowed recognition of these cases as adenocarcinoma of the lung. Figure 3A–C shows a digital CM image of a poorly differentiated adenocarcinoma with corresponding DQ-stained preparation of the imaged smear. The cytomorphological features of 3 smears and 4 cell pellets included high-grade tumor cells without clear evidence of three-dimensional clusters, indicating gland formation, as well as dense or delicate cytoplasm, suggesting squamous or glandular differentiation with overall features that could not be clearly recognized as squamous or adenocarcinoma of the lung. These cases were consistent with poorly differentiated non–small cell lung carcinoma. Two of the smears showed high cellularity with many single tumor cells, few loose clusters with stippled nuclear chromatin, granular cytoplasm, and eccentrically placed nucleus. The cytomorphological features in these cases allowed recognition as low-grade neuroendocrine tumor. Eight smears and 4 cell pellets showed high cellularity, including atypical spindle cells with moderate to severe nuclear atypia and mild to moderate nuclear pleomorphism with the presence of mitotic figures. A few necrotic fragments were also recognized in 4 of the cases, which indicated malignant spindle cell tumor.

### Kidney

The digital CM image of a kidney lesion showed many single cells and small clusters, including cells with uniform round nuclei and abundant granular cytoplasm. There was no evidence of atypia, such as mitotic figures, in the cells. The cytomorphological features allowed recognition of the case as consistent with oncocytoma. Thirteen smears and 4 cell pellets showed high cellularity associated with necrosis in 5 cases. The tumor cells showed varying grades of nuclear atypia, including prominent nucleolus, nuclear pleomorphism, and mitotic figures in many cases. The cytoplasm was variable in amount, delicate, and vacuolated, allowing recognition as conventional renal cell carcinoma. The cell pellets also clearly showed transgressing blood vessels associated with fragments of tumor cells. The overall cytomorphological features allowed recognition of the digital CM images of these smears and cell pellets prepared from kidney tumors as clear cell renal cell carcinoma.

### Liver

**Fig. 4:**
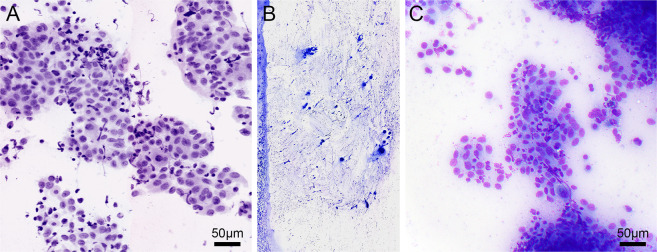
Digital confocal microscopy image of a smear from a liver specimen. Pseudocolored digital CM image of a smear showing poorly differentiated adenocarcinoma in liver (**A**). The cytomorphological features noted in the DQ-stained smear (**B**) are similar (**C**).

**Fig. 5:**
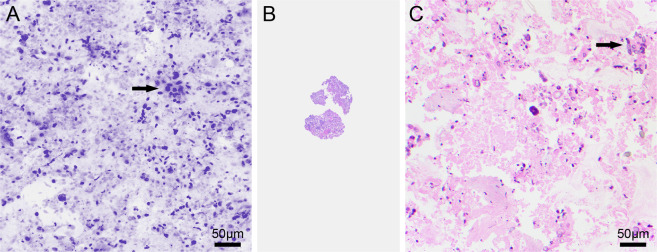
Digital confocal microscopy image of a cell pellet from a liver specimen. Pseudocolored digital CM image (**A**) of a cell pellet of a liver tumor showing rare clusters of poorly differentiated carcinoma in a background of extensive necrosis. The corresponding cell block (**B**) prepared from the imaged cell pellet shows similar features (**C**).

The digital CM images of 7 smears and 5 cell pellets showed abundant polygonal cells with a centrally placed nucleus and small nucleolus associated with abundant finely granular cytoplasm, allowing recognition as benign hepatocytes. The images in 17 smears and 4 cell pellets showed high cellularity associated with varying amounts of necrosis. The tumor cells were distributed as three-dimensional clusters and single cells, some with an elongated hyperchromatic nucleus and some with an epithelioid appearance. The nuclei of the tumor cells showed varying grades of atypia, including prominent nucleolus, mitotic figures, and scant to moderate amounts of cytoplasm. The overall cytomorphological features allowed recognition of these cases as adenocarcinoma. Figure 4A–C shows a digital CM image of a liver tumor that reveals poorly differentiated adenocarcinoma with the corresponding DQ-stained preparation of the imaged smear. The findings in 3 smears and 2 cell pellets showed extensive necrosis with few tumor cells. These cases could be recognized as few clusters of viable poorly differentiated carcinoma cells that were present amid the extensive necrosis. Figure 5A–C shows the digital CM image of a cell pellet with very few clusters of viable tumor cells associated with extensive necrosis and the corresponding H&E-stained cell block section.

## Discussion

The results of our study demonstrated for the first time the feasibility of using digital CM to evaluate cytological specimens. The acquisition of digital confocal images within 2–3 min that could be viewed at the site of procurement or remotely in real time or at any time exemplifies the potential utility of this imaging modality for rapid on-site evaluation of cytological specimens in real time at the time of procurement as well as for those types of specimens that do not require immediate evaluation. The preservation of the integrity of the cells for subsequent conventional preparation suggests that initial evaluation of the digital CM images can provide a choice based on the findings. All of the cytological material can be conserved to prepare a conventional cell block or can be immediately triaged for nucleic acid extraction and molecular testing. Alternately, the material can be retained as smears if cell block preparation for ancillary testing is not found to be necessary.

The only other optical imaging techniques that were used in the way we used CM for cytological preparations was FF-OCT and quantitative phase imaging (QPI). Grieve et al used FF-OCT to evaluate 24 endoscopic ultrasound−guided fine-needle aspiration and biopsy specimens obtained from the pancreas, stomach, and lymph nodes^7, 8^. The authors compared the results of FF-OCT with those of histopathological examination of the imaged cytological material and showed that FF-OCT imaging could be used as a rapid, noninvasive, and nondestructive modality for the evaluation of specimens. Of note, whereas abnormal architectural features could be identified with use of FF-OCT imaging, cellular details could not be appreciated, unlike with digital CM. More recently, Zhou et al. reported the study design of a prospective clinical study of 80 patients from a single center with the goal of evaluating the sensitivity and specificity of FF-OCT for differentiating various types of pancreatic diseases by using endoscopic ultrasound−guided fine-needle aspiration and biopsy specimens^9^. The results of the study, however, have not yet been reported. Pham et al performed QPI using unstained Thin-Prep prepared urine cytology slides prepared from 28 patients. They used this technique to calculate nuclear/cell dry mass, their entropy and nucleaus-to-cell mass ratio for several hundred cells per specimen and correlated the results with follow-up diagnoses^8^. The nuclear mass and its entropy of urothelial cells showed progressive increase across the different cytological categories and was significantly different between the negative and positive categories. While this imaging technique does not allow categorization based on conventional cytomorphological features unlike CM, it has the potential to serve as an ancillary test to improve the diagnostic accuracy of cytopathological specimens.

Evaluation of smears by CM essentially provides an alternate technique for visualization of cellular constituents that is similar to the currently used practice of staining smears with DQ or Papanicolaou stains for conventional cytopathological examination. Our ability to acquire the digital CM images rapidly, within 2–3 min, and the availability of digital CM images for interpretation suggests that CM images can serve the same purpose as stained smears for rapid on-site examination^10^. However, there are distinct advantages to using CM for evaluation of smears compared with the use of stained smears for cytopathological evaluation. Smears stained by DQ or Pap require a light microscope or must be digitized by using one of a variety of techniques for remote viewing. In contrast, since the CM images are inherently digital, they can be viewed immediately at the site of procurement or remotely without the need for a microscope or an added step of obtaining digital images of the stained smears. In addition, unlike the current practice, in which the cellular constituents of the stained smears must be either scraped or subjected to a process of cell transfer to lift the cells to the prepared cell block, the cellular constituents of the smear used for CM can be easily introduced to the RPMI solution for preparing a cell block soon after completion of imaging^11–13^. Also, the smear used for CM can be used immediately for molecular testing based on the quality of the smear, without the need to remove the cover slip and xylene and scraping the smear to retrieve the stained cells for nucleic acid extraction for molecular testing^14–16^.

Our findings also demonstrated the utility of CM for evaluating cell suspensions. We found that CM enables the evaluation of cells in RPMI suspension within minutes, which could permit pathologists to immediately determine the suitability of the cytological material to prepare a cell block. Otherwise, they must wait 6–24 h to examine H&E-stained sections of the cell block, at which point it may be too late to procure additional material to improve the quality of nondiagnostic or suboptimal specimens. The availability of high-quality cell blocks for performing ancillary testing is well recognized^17, 18^. Whereas smears as well as cell blocks can be used for molecular testing, cell blocks but not smears are optimal for both immunohistochemical testing of diagnostic markers and evaluating prognostic and predictive biomarkers for targeted therapy^19, 20^.

In current practice, the quality of the intended cell block preparation cannot be evaluated in real time at the bedside, when cytology specimens are procured and placed in fixative solutions. We used RPMI solution for collecting cells in suspensions. In ongoing studies, we are evaluating the suitability of digital CM for imaging cells collected in other fixative solutions such as Cytolyte, and preliminary findings have been promising. The availability of CM imaging can also be useful for conserving all of the cellular elements of the cytology specimen, unlike in the current practice of preparing smears for immediate assessment and collection of cells in rinse for preparation of the cell block.

The fact that CM can be used to examine smears and cell pellets has positive implications for cytopathology practice. The ability to evaluate smears and/or cell pellets rapidly for cytomorphological examination, together with the possibility of conserving all of the material for preparation of a cell block, are distinct advantages of digital CM imaging and can be game changers for medical practice. The importance of high-quality cell blocks cannot be overemphasized in the today’s era of personalized cancer treatment, particularly when concurrent tissue procurement based on core needle biopsy is either not performed or is suboptimal or nondiagnostic for determining the status of various biomarkers for targeted therapy of the tumor. The integrity of the procured material exposed to fluorescent dyes such as acridine orange for subsequent immunohistochemical and molecular testing has been demonstrated in previous reports with use of surgical pathology material^21, 22^. With use of technological advancements, the quality and pseudocoloring of the digital CM images that could be obtained from cytology specimens in our study, specifically the resolution and digital staining, can be further improved to appear much better than what was achieved in our study.

In essence, the rapid evaluation of cytology specimens with use of next-generation digital microscopy tools such as CM can bring revolutionary changes to the field of cytopathology. The results of our feasibility study clearly suggest the utility of CM for evaluating cytology specimens and warrant the validation of digital CM imaging in prospective clinical studies, currently ongoing in our clinical practice, for potential incorporation of this technique into cytopathology clinical practice.

## References

[1] Krishnamurthy S, Brown JQ, Iftimia N, Levenson RM, Rajadhyaksha M (2019). Ex vivo microscopy: a promising next-generation digital microscopy tool for surgical pathology practice. Arch. Pathol. Lab. Med..

[2] Peters N, Schubert M, Metzler G, Geppert JP, Moehrle M (2019). Diagnostic accuracy of a new ex vivo confocal laser scanning microscope compared to H&E-stained paraffin slides for micrographic surgery of basal cell carcinoma. J. Eur. Acad. Dermatol. Venereol..

[3] Hollon TC (2020). Near real-time intraoperative brain tumor diagnosis using stimulated Raman histology and deep neural networks. Nat. Med..

[4] Krishnamurthy S (2020). Comparison of real-time fluorescence confocal digital microscopy with hematoxylin-eosin-stained sections of core-needle biopsy specimens. JAMA Netw. Open.

[5] Marenco J. et al. Evaluation of fluorescent confocal microscopy for intraoperative analysis of prostate biopsy cores. *Eur. Urol. Focus.* 2020. 10.1016/j.euf.2020.08.013. Epub ahead of print. 10.1016/j.euf.2020.08.013 32912840

[6] Rocco B. et al. Digital biopsy with fluorescence confocal microscope for effective real-time diagnosis of prostate cancer: a prospective, comparative study. *Eur. Urol. Oncol.* 2020. 10.1016/j.euo.2020.08.009. Epub ahead of print. 10.1016/j.euo.2020.08.009 32952095

[7] Grieve K, Palazzo L, Dalimier E, Vielh P, Fabre M (2015). A feasibility study of full-field optical coherence tomography for rapid evaluation of EUS-guided microbiopsy specimens. Gastrointest. Endosc..

[8] Pham HV, Pantanowitz L, Liu Y (2016). Quantitative phase imaging to improve the diagnostic accuracy of urine cytology. Cancer Cytopathol..

[9] Zhou W (2020). Comparison of full-field optical coherence tomography imaging for pancreatic tissue sample obtained by EUS-fine-needle biopsy and conventional histological examination: a study protocol for a prospective trial. Endosc. Ultrasound.

[10] Lin O (2018). Telecytology for rapid on-site evaluation: current status. J. Am. Soc. Cytopathol..

[11] Bhatia P (2008). Cell blocks from scraping of cytology smear: comparison with conventional cell block. Acta Cytol.

[12] Gong Y, Joseph T, Sneige N (2005). Validation of commonly used immunostains on cell‐transferred cytologic specimens. Cancer Cytopathol..

[13] Wu HH, Jones KJ, Cramer HM (2013). Immunocytochemistry performed on the cell-transferred direct smears of the fine-needle aspirates: a comparison study with the corresponding formalin-fixed paraffin-embedded tissue. Am. J. Clin. Pathol..

[14] Knoepp SM, Roh MH (2013). Ancillary techniques on direct-smear aspirate slides: a significant evolution for cytopathology techniques. Cancer Cytopathol..

[15] Treece AL (2016). FNA smears as a potential source of DNA for targeted next-generation sequencing of lung adenocarcinomas. Cancer Cytopathol..

[16] Wu HH (2014). Utilization of cell-transferred cytologic smears in detection of EGFR and KRAS mutation on adenocarcinoma of lung. Mod. Pathol..

[17] Saqi A (2016). The state of cell blocks and ancillary testing: past, present, and future. Arch Pathol. Lab. Med..

[18] Shidham VB (2019). CellBlockistry: chemistry and art of cell-block making - a detailed review of various historical options with recent advances. Cytojournal.

[19] Jain D (2019). Immunocytochemistry for predictive biomarker testing in lung cancer cytology. Cancer Cytopathol..

[20] Roy-Chowdhuri S (2020). Immunocytochemistry of cytology specimens for predictive biomarkers in lung cancer. Transl Lung Cancer Res..

[21] Elfgen C (2019). Comparative analysis of confocal microscopy on fresh breast core needle biopsies and conventional histology. Diagn. Pathol..

[22] Cahill LC (2018). Rapid virtual hematoxylin and eosin histology of breast tissue specimens using a compact fluorescence nonlinear microscope. Lab. Invest..

